# Remote ischemic conditioning improves rat brain antioxidant defense in a time-dependent mechanism

**DOI:** 10.1590/ACB360707

**Published:** 2021-09-03

**Authors:** Andrew Moraes Monteiro, Rodrigo Paracampo Couteiro, Dora Fonseca da Silva, Sérgio Cunha Trindade, Renata Cunha Silva, Luís Fernando Freitas de Sousa, Deivid Ramos dos Santos, Jofre Jacob da Silva Freitas, Marcus Vinícius Henriques Brito

**Affiliations:** 1 MD. Laboratory for Experimental Surgery - Department of Experimental Surgery – Universidade do Estado do Pará (UEPA) – Belém (PA), Brazil.; 2 MD. Laboratory for Experimental Surgery - Department of Experimental Surgery – Universidade do Estado do Pará (UEPA) – Belém (PA), Brazil.; 3 MD. Laboratory for Experimental Surgery - Department of Experimental Surgery – Universidade do Estado do Pará (UEPA) – Belém (PA), Brazil.; 4 MS. Laboratory for Experimental Surgery - Department of Experimental Surgery – Universidade do Estado do Pará (UEPA) – Belém (PA), Brazil.; 5 OTS. Laboratory for Morphophysiology Applied to Health - Department of Experimental Surgery - Universidade do Estado do Pará (UEPA) – Belém (PA), Brazil.; 6 MS. Laboratory for Experimental Surgery - Department of Experimental Surgery – Universidade do Estado do Pará (UEPA) – Belém (PA), Brazil.; 7 MS. Laboratory for Experimental Surgery - Department of Experimental Surgery – Universidade do Estado do Pará (UEPA) – Belém (PA), Brazil.; 8 PhD. Laboratory for Morphophysiology Applied to Health - Department of Experimental Surgery – Universidade do Estado do Pará (UEPA) – Belém (PA), Brazil.; 9 PhD. Laboratory for Experimental Surgery - Department of Experimental Surgery – Universidade do Estado do Pará (UEPA) – Belém (PA), Brazil.

**Keywords:** Ischemia, Reperfusion Injury, Ischemic Postconditioning, Stroke, Antioxidants, Rats

## Abstract

**Purpose:**

To clarify the best protocol for performing remote ischemic conditioning and to minimize the consequences of ischemia and reperfusion syndrome in brain, the present study aimed to evaluate different time protocols and the relation of the organs and the antioxidant effects of this technique.

**Methods:**

The rat’s left femoral artery was clamped with a microvascular clamp in times that ranged from 1 to 5 minutes, according to the corresponding group. After the cycles of remote ischemic conditioning and a reperfusion of 20 minutes, the brain and the left gastrocnemius were collected. The samples were used to measure glutathione peroxidase, glutathione reductase and catalase levels.

**Results:**

In the gastrocnemius, the 4-minute protocol increased the catalase concentration compared to the 1-minute protocol, but the latter increased both glutathione peroxidase and glutathione reductase compared to the former. On the other hand, the brain demonstrated higher catalase and glutathione peroxidase in 5-minute group, and the 3-minute group reached higher values of glutathione reductase.

**Conclusions:**

Remote ischemic conditioning increases brain antioxidant capacity in a time-dependent way, while muscle presents higher protection on 1-minute cycles and tends to decrease its defence with longer cycles of intermittent occlusions of the femoral artery.

## Introduction

Ischemia and reperfusion injury (I/R) occur when the blood flow is interrupted to an organ or tissue and, after a certain time, it is reestablished. The reperfusion injury is an important factor that triggers a variety of pathophysiological processes, such as a stroke^1^. During the process of I/R damage, a cascade of pathological events leads to excitotoxicity, inflammatory response, and the production of reactive oxygen species (ROS), which causes multiple and progressive damages, such as lipid peroxidation and mitochondrial injury^2^.

Furthermore, a new technique called remote ischemic conditioning has recently been described in order to reduce the consequences of oxidative stress caused by I/R injury. Such procedure consists of repeated cycles of ischemia and reperfusion, which can be applied prior to the ischemia (preconditioning)^3^, during the ischemia (perconditioning)^4^ or after the ischemia (postconditioning)^5^. In addition, remote ischemic conditioning is considered an important protective therapy to brain tissue^6^^,^ 7, as well as muscle tissue, which can be protected from damage of the I/R syndrome such as rhabdomyolysis^8^ and raise the levels of antioxidant defense such as catalase (CAT), glutathione peroxidase (GPx) and glutathione reductase (GR).

Another important fact is the role of enzymes in this oxidative stress. GPx and CAT are particularly noteworthy, since they are degrading agents (H_2_O_2_) and have a high antioxidant effect^9^. During I/R damage, maintenance of GPx levels is a protective measure against ROS^10^ and implies improved blood flow through angiogenesis^11^. It is worth noticing the importance of GR in the maintenance of GPx levels, so that they do not rise in a cytotoxic or in a deregulated manner^12^. Finally, CAT has a similar function that may be a signaling factor not only of I/R syndrome, but also of several pathologies, such as metabolic disorders and hypertension^13^.

In this regard, there are many protocols of conditioning time, especially in relation to the brain^6^ and muscles^14^^,^ 15. Therefore, in order to clarify the best protocol for performing remote ischemic conditioning (RIC) and to minimize the consequences of I/R syndrome in brain, the present study aimed to evaluate different time protocols and the relation of the organs and the antioxidant effects of this technique.

## Methods

All experiments were performed in accordance with the Brazilian law for scientific use of animals (Law No. 11.794/08) and the National Institutes of Health (NIH) guide for the care and use of laboratory animals (NIH Publications No. 8,023, revised 1978). The research was approved by the Animal Care and Use Committee of Universidade do Estado do Pará (No. 31/2017).

Forty female Wistar rats (10-12 weeks), weighing 250-300 g, were obtained from Instituto Evandro Chagas. The animals were maintained at individual cages, at 22°C, under a 12-hour light/dark cycle and allowed free access to water and standard chow. All surgical procedures and analyses were performed at the Laboratory of Morphophysiology Applied to Health.

### Anesthesia

The animals were anesthetized using an intraperitoneal injection of ketamine hydrochloride 10% (70 mg/kg) and xylazine hydrochloride 2% (10 mg/kg).

### Surgical procedures

After anesthetic induction, animals were placed in supine position. A 25-mm long skin incision was made in the left medial thigh, and the skeletal muscle was retracted to expose the femoral triangle and its neurovascular bundle. Then, the femoral artery was carefully dissected from femoral vein and surrounding tissue under a microscope DF Vasconcellos® magnification (x16)^16^^,^ 17.

### Remote ischemic conditioning protocol

RIC protocol consisted of alternating cycles of IR by clamping the left femoral artery with a microvascular clamp, and the times were 1, 2, 3, 4 and 5 minutes^17^^,^ 18. After the reperfusion time of 20 minutes, the animals were euthanized by decapitation^19^. Then, the brain and the left gastrocnemius were collected at the same time for the biochemical analysis.

### Experimental groups

The animals (N = 40) were distributed into the following six experimental groups:

- Control group (CG): the animals were submitted to a vascular dissection in the left femoral artery, but they were not submitted to any ischemic conditioning (n=5 rats);
- RIC-1: the animals were submitted to a vascular dissection in the left femoral artery and to three cycles of alternated ischemia and reperfusion of 1 minute each (n=7 rats);
- RIC-2: the animals were submitted to a vascular dissection in the left femoral artery and to three cycles of alternated ischemia and reperfusion of 2 minutes each (n=7 rats);
- RIC-3: the animals were submitted to a vascular dissection in the left femoral artery and to three cycles of alternated ischemia and reperfusion of 3 minutes each (n=7 rats);
- RIC-4: the animals were submitted to a vascular dissection in the left femoral artery and to three cycles of alternated ischemia and reperfusion of 4 minutes each (n=7 rats);
- RIC-5: the animals were submitted to a vascular dissection in the left femoral artery and to three cycles of alternated ischemia and reperfusion of 5 minutes each (n=7 rats).

**Figure 1:**
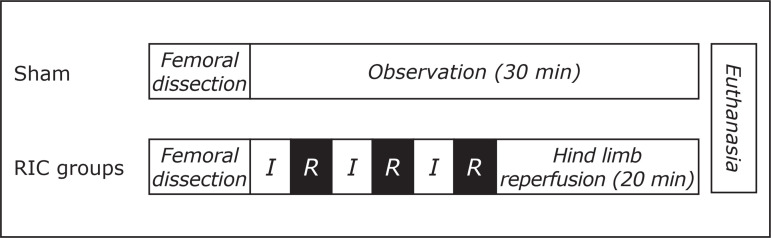
Experimental design.

After femoral dissection, all RIC groups were submitted to alternating cycles of ischemia and reperfusion, whose times ranged from 1 to 5 minutes, followed by 20 minutes of hind limb reperfusion. Sham group was submitted only to femoral dissection and 30-minute observation. At the end of either observation or reperfusion, euthanasia was performed (Fig. 1).

### Biochemical analysis

The samples were homogenized in saline solution and then immediately centrifuged at 3,000 rpm for 10 minutes. After centrifugation, samples were directly transferred to Eppendorf tubes and stored at -80°C until assayed. GPx (mIU/mL), GR (mIU/mL) and CAT (IU/mL) levels were determined. GPx and GR activity were measured by following the changes in nicotinamide adenine dinucleotide phosphate (NADPH) absorbance at 340 nm. CAT was measured by the decomposition rate of H_2_O_2_ in the sample at 230 nm. To calculate GPx, GR, and CAT activities, extinction coefficient values established for H_2_O_2_ and NADPH were used.

### Statistical analysis

Statistical analysis was performed using the software BioEstat 5.3. All data were expressed as means standard ± deviation. Kolmogorov-Smirnov test was applied to confirm Gaussian distribution of the data. One-way analysis of variance with Tukey’s post hoc test was used to assess differences between groups. Kruskal-Wallis, followed by Dunn’s test, was used to analyze CAT concentration in gastrocnemius. Statistical significance was considered at p < 0.05.

## Results

**Figure 2:**
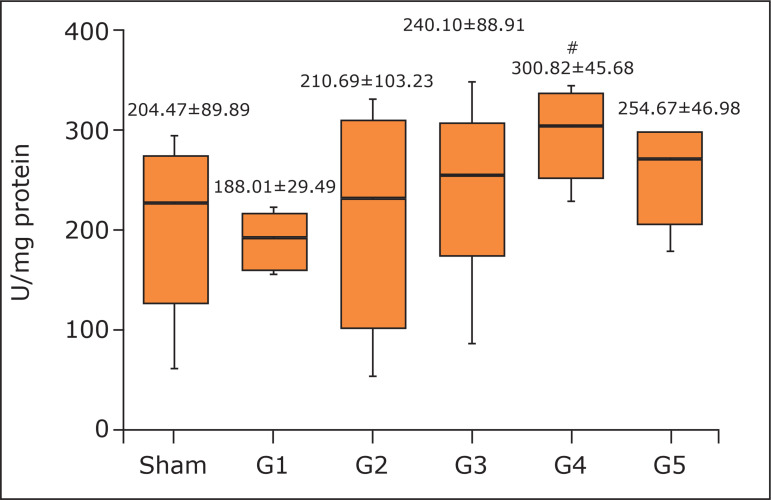
Concentration of catalase in the gastrocnemius muscle. Kruskal-Wallis, Dunn’s post hoc test, non-parametric distribution. Mean and standard deviation.

**Figure 3:**
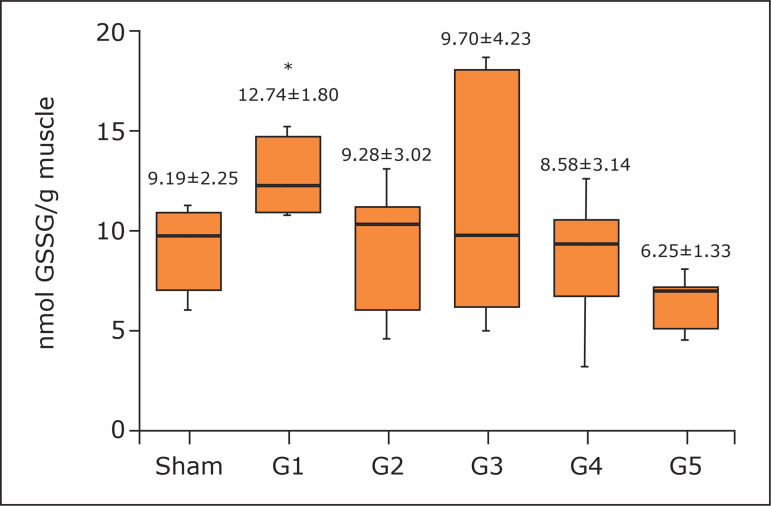
Concentration of glutathione peroxidase in milligrams of protein in the gastrocnemius muscle. One-way analysis of variance, Tukey’s post hoc test. Mean and standard deviation.

**Figure 4:**
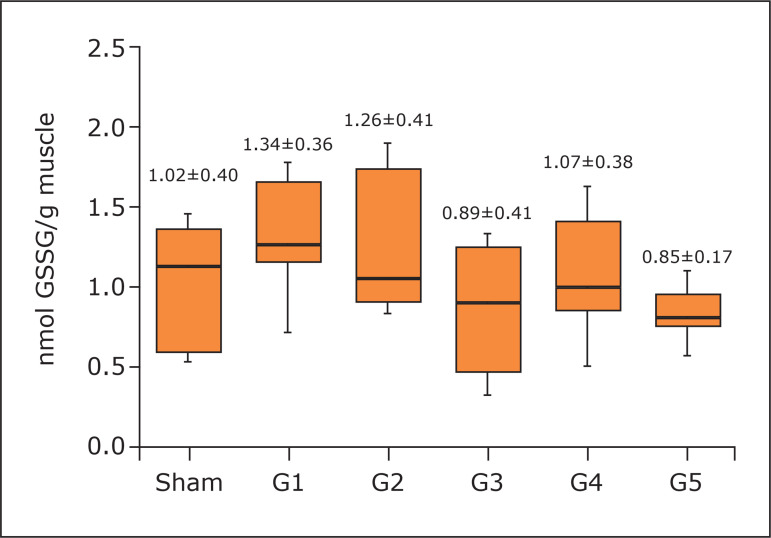
Concentration of glutathione reductase in the gastrocnemius muscle. One-way analysis of variance, Tukey’s post hoc test. Mean and standard deviation. No statistical difference (p = 0.0996).

No animal died during the anesthesia, procedures, or reperfusion period. The 4-minute (G4) protocol increased gastrocnemius CAT concentration (300.82±45.68) compared to the 1-minute (G1) protocol (188.01±29.49; p < 0.05) of RIC (Fig. 2). The 1-minute protocol increased both GPx (12.74±1.80; p < 0.01 G1 *versus* G5) (Fig. 3) and GR (1.34±0.36; no statistical difference; p = 0.0996) in the gastrocnemius muscle (Fig. 4).

*p < 0.05 G4 *vs*. G1.

*p < 0.01 G1 *vs.* G5.

**Figure 5:**
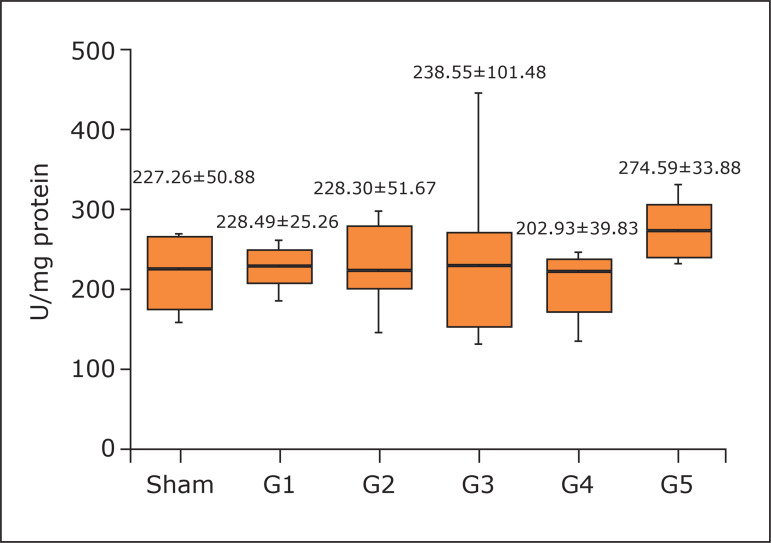
Concentration of catalase in the brain. One-way analysis of variance, Tukey’s post hoc test. Mean and standard deviation. No statistical difference (p = 0.0533).

**Figure 6:**
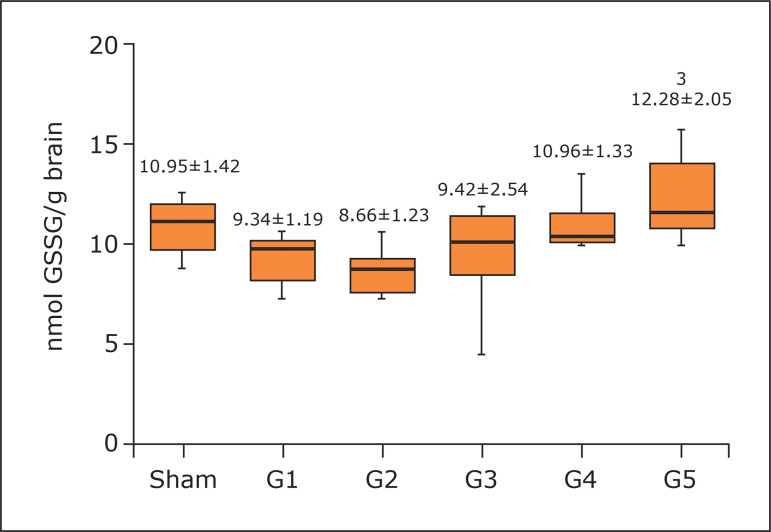
Concentration of glutathione peroxidase in the brain. One-way analysis of variance, Tukey’s post hoc test. Mean and standard deviation.

**Figure 7:**
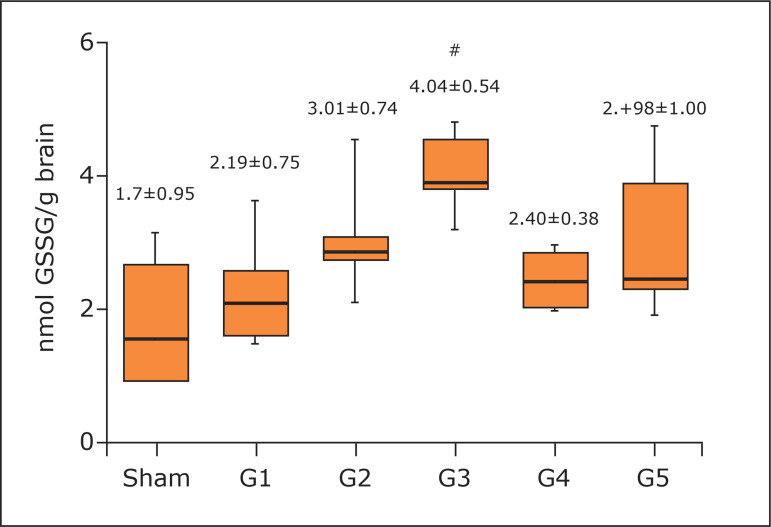
Concentration of glutathione reductase in the brain. One-way analysis of variance, Tukey’s post hoc test. Mean and standard deviation.

Regarding brain antioxidant activity, G5 presented higher CAT (274.59±33.88; no statistical difference; p = 0.0533) and GPx (12.28±2.05; p < 0.05 G5 *vs.* G1 and G3, p < 0.01 G5 *vs.* G2) concentrations (Figs. 5 and 6). However, the 3-minute protocol reached higher values of GR (4.04±0.54) when compared to sham group, G1 and G4 (p < 0.01) (Fig. 7).

*p < 0.05 G5 *vs*. G1 and G3, p < 0.01 G5 *vs*. G2

*p < 0.01 G3 *vs*. sham, G1 and G4.

## Discussion

The muscle antioxidant capacity tends to reduce with the increase of the duration of cycles. We hypothesized that muscle of hind limbs, such as gastrocnemius, are submitted directly to the effects of arterial occlusion. There is elevation on enzymatic concentrations of GPx and GR in shorter cycles and possibly saturation of RIC repercussion in longer protocols.

On the other hand, the most of antioxidant enzymes in brain reached greater concentration with a 5-minute protocol, what suggests that brain antioxidant defense is elicited by RIC in a time-dependent way. It is influenced by the duration of cycles of occlusion and reperfusion on the hide limb.

The different patterns observed in the muscle and in remote organs are possibly related to the underlying mechanisms of RIC. Longer cycles are necessary to activate different pathways supposed to be related to this technique and to evoke a protective effect in distant tissues, for instance, a neural pathway, in which sublethal ischemic stimulus provides an afferent signal to the central nervous system^16^^,^ 20 ^,^ 21. Consequently, there is an efferent response, through activation of parasympathetic nerves, that plays a role in modulating vascular activity and increasing anti-inflammatory substances^16^.

This neurogenic pathway was studied by Czigány *et al*.^17^ in a model of hepatic ischemia and reperfusion. They showed that hepatoprotection elicited by perconditioning, demonstrated in some studies^1^^,^ 22 ^,^ 23, was abolished after femoral and sciatic nerve resection.

Another mechanism is proposed to explain how brief cycles of IR improves antioxidant capacity distantly. Some authors suggest that humoral factors are released from the tissue submitted to intermittent vascular occlusion, as adenosine, bradykinin and opioids^24^. Thus, the activation of those effector signals allow interaction between remote organs and the hind limb, and starts intracellular response, for example activation of RISK and SAFE pathways^25^.

The protocol using three alternating cycles of 5 minutes of ischemia and reperfusion was chosen, because it was extensively studied in perconditioning research^1^^,^ 26 ^-^ 29, showing promisors results in hepatic, renal and cardiac IR. Costa *et al*.^30^, applying the RIC in hind limb of rats without inducing IR injury, showed a temporary increasing on renal and hepatic total antioxidant capacity 10 minutes after its use. Those data provided evidence that remote techniques elevate total amount of reducing substances even in the absence of aggression mechanism.

Our results can contribute to stablish better experimental protocols to induce brain protection using per and remote postconditioning, in view of the existence of many different protocols using a wide range of times^14^ in cerebrovascular research, as well as to clarify the enzymatic pattern behind intermittent occlusion. Thus, further studies are needed to evaluate protocols using longer intervals and different number of cycles to reach maximum protective effect against brain ischemia in animal models.

Regarding the limitations of our research, an increasing in antioxidant capacity is not the only effect expected with the application of RIC. Increased transcription of antiapoptotic proteins, activation of RISK and SAFE pathways, nitric oxide synthase activity, release of nitric oxide, and vasomotor effects are variables that change with RIC, but they were not analyzed in the present study. New investigations can clarify RIC’s role on these variables. Furthermore, statistical significance on the levels of muscle GR and brain CAT could be reached in larger series.

## Conclusion

RIC increases brain antioxidant capacity in a time-dependent way, while muscle presents higher protection on the 1-minute cycles and trends to decrease its defense with longer cycles of intermittent occlusion of femoral artery.

## References

[1] Lou Z, Wang AP, Duan XM, Hu GH, Song GL, Zuo ML, Yang ZB (2018). Upregulation of NOX2 and NOX4 mediated by TGF-β signaling pathway
exacerbates cerebral ischemia/reperfusion oxidative stress
injury. Cell Physiol Biochem.

[2] Ribeiro RFG, Couteiro RP, Monteiro AM, Rodrigues IADS, Cavalcante LCDC, Gouveia EHH, Galvão LN, Lopes LRO, Yasojima EY, Brito MVH (2017). Perconditioning associated to hypertonic saline solution on liver
function improvement after ischemia/reperfusion injury. Acta Cir Bras.

[3] Murry CE, Jennings RB, Reimer KA. (1986). Preconditioning with ischemia: a delay of lethal cell injury in
ischemic myocardium. Circulation.

[4] Consegal M, Núñez N, Barba I, Benito B, Ruiz-Meana M, Inserte J, Ferreira-González I, Rodríguez-Sinovas A. (2021). Citric acid cycle metabolites predict infarct size in pigs
submitted to transient coronary artery occlusion and treated with succinate
dehydrogenase inhibitors or remote ischemic perconditioning. Int J Mol Sci.

[5] Zhao ZQ, Corvera JS, Halkos ME, Kerendi F, Wang NP, Guyton RA, Vinten-Johansen J. (2003). Inhibition of myocardial injury by ischemic postconditioning
during reperfusion: comparison with ischemic preconditioning. Am J Physiol Heart Circ Physiol.

[6] Ren C, Li S, Wang B, Han R, Li N, Gao J, Li X, Jin K, Ji X (2018). Limb remote ischemic conditioning increases Notch signaling
activity and promotes arteriogenesis in the ischemic rat
brain. Behav Brain Res.

[7] Ma J, Ma Y, Dong B, Bandet MV, Shuaib A, Winship IR (2017). Prevention of the collapse of pial collaterals by remote ischemic
perconditioning during acute ischemic stroke. J Cereb Blood Flow Metab.

[8] Yin TC, Wu RW, Sheu JJ, Sung PH, Chen KH, Chiang JY, Hsueh SK, Chung WJ, Lin PY, Hsu SL, Chen CC, Chen CY, Shao PL, Yip HK (2018). Combined therapy with extracorporeal shock wave and
adipose-derived mesenchymal stem cells remarkably improved acute
ischemia-reperfusion injury of quadriceps muscle. Oxid Med Cell Longev.

[9] Glorieux C, Zamocky M, Sandoval JM, Verrax J, Calderon PB. (2015). Regulation of catalase expression in healthy and cancerous
cells. Free Radic Biol Med.

[10] Teixeira RKC, Costa FLDS, Calvo FC, Santos DRD, Yasojima EY, Brito MVH (2019). effect of copaiba oil in intestinal mucosa of rats submitted to
hypovolemic shock. Arq Bras Cir Dig.

[11] Watanabe Y, Murdoch CE, Sano S, Ido Y, Bachschmid MM, Cohen RA, Matsui R. (2016). Glutathione adducts induced by ischemia and deletion of
glutaredoxin-1 stabilize HIF-1α and improve limb
revascularization. Proc Natl Acad Sci U S A.

[12] Kedrowski BL, Gutow JH, Stock G, Smith M, Jordan C, Masterson DS (2014). Glutathione reductase activity with an oxidized methylated
glutathione analog. J Enzyme Inhib Med Chem.

[13] McLeod SL, Iansavichene A, Cheskes S (2017). Remote ischemic perconditioning to reduce reperfusion injury
during acute st-segment-elevation myocardial infarction: a systematic review
and meta-analysis. J Am Heart Assoc.

[14] Chen G, Thakkar M, Robinson C, Doré S (2018). Limb remote ischemic conditioning: mechanisms, anesthetics, and
the potential for expanding therapeutic options. Front Neurol.

[15] Mase VJ, Roe JL, Christy RJ, Dubick MA, Walters TJ (2016). Postischemic conditioning does not reduce muscle injury after
tourniquet-induced ischemia-reperfusion injury in rats. Am J Emerg Med.

[16] Lim SY, Yellon DM, Hausenloy DJ. (2010). The neural and humoral pathways in remote limb ischemic
preconditioning. Basic Res Cardiol.

[17] Czigány Z, Turóczi Z, Kleiner D, Lotz G, Homeyer A, Harsányi L, Szijártó A (2015). Neural elements behind the hepatoprotection of remote
perconditioning. J Surg Res.

[18] Yamaki VN, Gonçalves TB, Coelho JV, Pontes RV, Costa FL, Brito MV (2012). Protective effect of remote ischemic per-conditioning in the
ischemia and reperfusion-induce renal injury in rats. Rev Col Bras Cir..

[19] Kongara K, McIlhone AE, Kells NJ, Johnson CB (2014). Electroencephalographic evaluation of decapitation of the
anaesthetized rat. Lab Anim.

[20] Zhang Y, Zhang X, Chi D, Wang S, Wei H, Yu H, Li Q, Liu B. (2016). Remote ischemic preconditioning for prevention of acute kidney
injury in patients undergoing on-pump cardiac surgery: a systematic review
and meta-analysis. Medicine (Baltimore).

[21] Liu Z, Zhao Y, Lei M, Zhao G, Li D, Sun R, Liu X (2021). Remote ischemic preconditioning to prevent acute kidney injury
after cardiac surgery: a meta-analysis of randomized controlled
trials. Front Cardiovasc Med.

[22] Li DY, Liu WT, Wang GY, Shi XJ. (2018). Impact of combined ischemic preconditioning and remote ischemic
perconditioning on ischemia-reperfusion injury after liver
transplantation. Sci Rep.

[23] Costa FL, Yamaki VN, Gonçalves TB, Coelho JV, Percário S, Brito MV. (2014). Combined remote ischemic perconditioning and local
postconditioning on liver ischemia-reperfusion injury. J Surg Res.

[24] Yamaguchi T, Izumi Y, Nakamura Y, Yamazaki T, Shiota M, Sano S, Tanaka M, Osada-Oka M, Shimada K, Miura K, Yoshiyama M, Iwao H (2015). Repeated remote ischemic conditioning attenuates left ventricular
remodeling via exosome-mediated intercellular communication on chronic heart
failure after myocardial infarction. Int J Cardiol.

[25] Yamaki IN, Pontes RV, Costa FL, Yamaki VN, Teixeira RK, Yasojima EY, Brito MV (2016). Kidney ischemia and reperfunsion syndrome: effect of lidocaine
and local postconditioning. Rev Col Bras Cir.

[26] Costa FLS, Yamaki VN, Teixeira RKC, Feijó DH, Valente AL, Carvalho LTF, Yasojima EY, Brito MVH (2017). Perconditioning combined with postconditioning on kidney ischemia
and reperfusion. Acta Cir Bras.

[27] Oliveira RC, Brito MV, Ribeiro RF, Oliveira LO, Monteiro AM, Brandão FM, Cavalcante LC, Gouveia EH, Henriques HY (2017). Influence of remote ischemic conditioning and tramadol
hydrochloride on oxidative stress in kidney ischemia/reperfusion injury in
rats. Acta Cir Bras.

[28] Brito MV, Yasojima EY, Percário S, Ribeiro RF, Cavalcante LC, Monteiro AM, Couteiro RP, Rodrigues IA, Santos HA (2017). Effects of hypertonic saline solution associated to remote
ischemic perconditioning in kidney ischemia/reperfusion injury in
rats. Acta Cir Bras.

[29] Makkos A, Szántai Á, Pálóczi J, Pipis J, Kiss B, Poggi P, Ferdinandy P, Chatgilialoglu A, Görbe A (2020). A comorbidity model of myocardial ischemia/reperfusion injury and
hypercholesterolemia in rat cardiac myocyte cultures. Front Physiol.

[30] Costa FL, Teixeira RK, Yamaki VN, Valente AL, Silva AM, Brito MV, Percário S (2016). Remote ischemic conditioning temporarily improves antioxidant
defense. J Surg Res.

